# A Recovery-Oriented Suicide Prevention Program Led by Peer Specialists for Veterans With Serious Mental Illness: Protocol for a Pilot Randomized Controlled Trial

**DOI:** 10.2196/66182

**Published:** 2025-08-20

**Authors:** Samantha Chalker, Jillian Carter, Yuki Imai, Colin Depp, Matthew Chinman

**Affiliations:** 1 VA San Diego Healthcare System San Diego, CA United States; 2 University of California, San Diego San Diego, CA United States; 3 VA Pittsburgh Healthcare System Pittsburgh, PA United States; 4 RAND Corporation Santa Monica, CA United States

**Keywords:** peers, peer support, recovery, suicide prevention, veterans, serious mental illness

## Abstract

**Background:**

Veterans with serious mental illness (SMI) have significantly elevated suicide risk compared to those without SMI. This group may also experience cognitive and functional impairments that complicate suicide prevention care.

**Objective:**

We detail a pilot randomized controlled trial protocol evaluating a novel peer specialist–delivered suicide prevention intervention tailored to SMI called Suicide Prevention by Peers Offering Recovery Tactics (SUPPORT). In SUPPORT, peer specialists are trained in foundational suicide prevention information and intervention delivery. Once a week for 50 minutes, a peer specialist meets with a veteran via telehealth or in person to deliver the 4 SUPPORT modules that focus on recovery and suicide prevention. The peer specialist conducts 2 check-in calls at 2 and 4 weeks after the final module to reinforce the intervention material and offer additional resources. Aim 1 will evaluate the feasibility and acceptability of SUPPORT. Aim 2 will determine the preliminary impact of SUPPORT versus enhanced standard care on personal recovery and suicide ideation severity (primary outcomes), as well as domains of veteran functioning (secondary outcomes). Standard care is enhanced such that all veterans receive additional suicide risk assessment, which may result in additional clinical interaction.

**Methods:**

A pilot randomized controlled trial with 50 veterans randomized into 2 groups will be conducted. Participants will be recruited from the Veterans Affairs San Diego Healthcare System. Veterans with SMI and past-1-month active suicide thoughts or past-3-month suicide behavior as defined by the Columbia–Suicide Severity Rating Scale will be included. Enrolled veterans will be randomized to receive SUPPORT or enhanced standard care. All veteran participants will be assessed at baseline and the 1-, 2-, and 3-month follow-up assessments. Feasibility is defined as attainment of recruitment goals and a ≥70% retention rate. Acceptability for veterans is measured using the Client Satisfaction Questionnaire–8. Acceptability for peer specialists is measured using a satisfaction question rated on a 5-point Likert scale. Primary outcomes are measured using the Recovery Assessment Scale–41 (personal recovery), as well as the Beck Scale for Suicide Ideation and the Columbia–Suicide Severity Rating Scale (suicide ideation severity). Secondary outcomes are measured using the Specific Level of Functioning Scale (functioning), the brief version of the World Health Organization Quality of Life Scale (quality of life), and a laboratory-developed self-knowledge task (suicide prevention strategy recall). Outcomes will be tested using mixed-effects models. Safety procedures for all participants are discussed.

**Results:**

This trial was funded in November 2022. Community-engaged intervention and protocol refinement were completed in January 2025. Data collection for the randomized controlled trial began in January 2025.

**Conclusions:**

By combining training for peer specialists with a novel recovery-oriented suicide prevention intervention, SUPPORT helps establish a role for peer specialists in suicide prevention and addressing suicide in veterans with SMI.

**Trial Registration:**

ClinicalTrials.gov NCT05537376; https://clinicaltrials.gov/study/NCT05537376

**International Registered Report Identifier (IRRID):**

DERR1-10.2196/66182

## Introduction

### Background

Among the veteran population, those with a serious mental illness (SMI), particularly veterans with bipolar disorder or schizophrenia spectrum disorders, are at markedly elevated suicide risk compared to veterans without SMI and nonveterans with SMI [1]. Making suicide prevention care challenging, people with SMI are often excluded from suicide-related treatment options [2,3]. There are also different means of suicide used, such as self-stabbing [4], as well as functional and cognitive impairments that may alter uptake of suicide prevention strategies in this group [5,6]. To address these unmet needs, we provide the rationale, design, and clinical pilot randomized controlled trial protocol for a novel augmentative peer-delivered, recovery-oriented suicide prevention intervention for veterans with SMI.

Several psychosocial rehabilitation initiatives in the Veterans Health Administration (VHA) have included individuals with lived experience (“peers,” now known as “peer specialists”) to support veterans with a wide range of mental health experiences [7]. In the context of VHA mental health care, peer specialists are defined as veterans with lived experience in recovery [8] from mental illness who are specifically trained, certified, and employed [9] to share their recovery story to support other veterans also experiencing mental illness [10,11]. Numbering approximately 1400 in VHA in 2024, peer specialists play an adjunctive yet integral role in VHA health care [7,12]. However, there is a dearth of existing interventions that use peer support services in suicide prevention initiatives [13-15] Reviews of this literature have concluded that the use of peer specialists for suicide prevention is feasible [13,14]. VHA peer specialist administrative data show that 8% are already working directly with veterans at high risk of suicide [16]. Moreover, peer support has been shown to be a positive adjunct to VHA suicide prevention initiatives by filling an existing gap [17]. Although there is limited suicide prevention training for peer specialists, VHA peer specialists report interest in receiving such specific training [11,18]. Thus, there is a tremendous opportunity to involve peer specialists in VHA suicide prevention initiatives.

### Objectives

The Suicide Prevention by Peers Offering Recovery Tactics (SUPPORT) program was co-developed by peer specialists and veterans with SMI along with scientific experts in the field [18]. The SUPPORT program includes training tailored to a peer’s scope of practice in suicide prevention (SUPPORT 101) and a recovery-oriented suicide prevention intervention (SUPPORT intervention) that was designed for and tailored to the unique experiences of veterans with SMI. This paper describes a protocol for a pilot randomized controlled trial aiming to preliminarily evaluate this peer specialist-delivered suicide prevention intervention program for veterans with SMI. The specific aims of this pilot study are as follows:

1. To evaluate the feasibility and acceptability of SUPPORT versus enhanced standard care. We hypothesize that SUPPORT will be feasible to deliver, as measured through attainment of recruitment goals and retention rates of ≥70%, with high satisfaction ratings.
2. To determine the preliminary impact of SUPPORT compared to enhanced standard care on personal recovery, suicide ideation severity, and domains of veteran functioning to determine whether a larger trial is warranted. We hypothesize that SUPPORT compared to enhanced standard care will yield greater enhancement of personal recovery and reduction in suicide ideation severity (primary outcomes), as well as enhanced psychosocial functioning, quality of life, and suicide prevention strategy recall.

## Methods

### Setting

This pilot randomized controlled trial is taking place in the Veterans Affairs (VA) San Diego Healthcare System. Recruitment sites are the medical center and the community-based outpatient clinics providing behavioral health care services.

### Ethical Considerations

The SPIRIT (Standard Protocol Items: Recommendations for Interventional Trials) reporting guidelines for clinical trials were used [19]. The trial was registered on ClinicalTrials.gov (NCT05537376; see Multimedia Appendix 1). This protocol was approved by the VA San Diego Healthcare System institutional review board (H210132). The institutional review board requires regular updates on the status of the project, including the number of participants enrolled, adverse events or unanticipated problems, number of withdrawals from the project, complaints about the research, and any protocol changes. The institutional review board also audits the consent forms quarterly and ensures that appropriate HIPAA (Health Insurance Portability and Accountability Act) information is included, with full regulatory audits taking place every 3 years. Taken together, the use of these methods allows for adequate evaluation of and response to the data and safety monitoring of both potential enrollees and actual participants.

All participants will provide written informed consent, as detailed in the Recruitment, Screening, and Enrollment Procedures section. It is clear in the consent form that SUPPORT is the experimental condition that is being added to the standard care that a veteran would receive. As part of informed consent, participants are told that they may withdraw from the study at any point without penalty, VHA will cover any care if harm occurs as part of their participation, and veteran participants randomized to SUPPORT may drop out of the intervention and continue with research assessments. Data collected are stored separately from protected health information, including electronic data that are only stored on the VHA network and are password protected, whereas hard copy data remain on VHA property behind 2 locks. Data are only accessible by those approved on the institutional review board protocol. Veteran participants are compensated with up to US $260 for completing all research assessments. Peer specialist participants are not eligible for monetary compensation due to their VHA employment and the federal funding mechanism; they are delivering the intervention as part of their regular tour of duty. Due to the nature of peer services being augmentative, all veteran participants are encouraged to maintain their other ongoing behavioral health care.

### Participants

#### Peer Specialist Participants

All VA San Diego Healthcare System–employed certified peer specialists are potentially eligible to be recruited into the study. All local peer specialists will be invited to the SUPPORT training as a service to the medical center per conversations with local leadership, although they must meet inclusion criteria to deliver the SUPPORT intervention. All VHA employees are required to maintain basic competencies in suicide prevention screening and referral procedures [20]. Thus, study inclusion criteria are (1) a minimum of 2 years of experience after certification as a peer specialist and (2) provision of verbal or written consent by the peer specialist for research staff to discuss their ability to participate with their direct clinical supervisor. The peer specialist’s direct clinical supervisor must agree to allow the peer specialist time as part of their current scope of practice to (1) participate in the 8-hour SUPPORT training, (2) participate in the study’s biweekly group supervision, and (3) take on at least one SUPPORT veteran participant at a given time.

#### Veteran Participants

The inclusion criteria are purposefully broad and include veterans who report active suicide ideation in the previous month (ie, Columbia–Suicide Severity Rating Scale, C-SSRS [21], score of ≥2) or suicide behavior in the previous 3 months. Additional inclusion criteria are (1) current SMI diagnosis (ie, “a mental, behavioral, or emotional disorder resulting in serious functional impairment, which substantially interferes with or limits one or more major life activities” [22]) and (2) capability to provide informed consent (via the University of California, San Diego, Brief Assessment of Capacity to Consent [23]). Prioritized SMI diagnoses include those with psychosis or bipolar symptoms. Veterans with a diagnosis that falls under the broad VHA definition of SMI, which includes the following—major depressive disorder, schizophrenia spectrum and other psychotic disorders, bipolar disorders, obsessive-compulsive disorder, panic disorder, posttraumatic stress disorder, and borderline personality disorder [24]—are eligible. To prioritize those with psychosis and bipolar symptomology, recruitment will be expanded to the broad SMI diagnoses if no veterans meet the criteria for psychosis-related or bipolar disorders, with a guideline that 4 out of every 5 veterans recruited should experience psychosis or bipolar symptoms.

The following are the exclusion criteria: (1) inability to complete the assessment battery, (2) current intoxication requiring immediate detoxification or an outpatient plan directed specifically to residential substance use disorder (not mental health) services, and (3) imminent psychiatric hospitalization. We will include participants with an active substance use disorder if they do not require acute detoxification or residential treatment, and they can participate after acute detoxification. Participants can be engaged in other forms of treatment, including psychotherapy, as SUPPORT is meant to augment existing treatment as is standard of peer services. Veterans who participated in the previous open pilot trial will not be eligible to participate in the pilot randomized controlled trial to allow for the intervention to reach a broad range of veterans.

### Recruitment, Screening, and Enrollment Procedures

#### Peer Specialist Participants

Research staff presented to the local peer specialists during their monthly team meeting to introduce the study. Peer specialists who met eligibility criteria were invited to follow up with research staff if they were interested in participating and instructed to provide verbal or written consent for research staff to contact their direct clinical supervisor. Next, the research staff contacted the peer specialists’ direct clinical supervisors to discuss inclusion criteria. If eligibility was determined, they were consented and enrolled in the study. If they were not eligible, they were informed and still invited to participate in the SUPPORT trainings.

#### Veteran Participants

The veteran recruitment processes were designed to produce minimal impact to usual care. The primary recruitment locations for this study include VHA inpatient and outpatient behavioral health clinics, emergency services, and specialty services (eg, suicide prevention coordinators) across the VA San Diego Healthcare System. Potential veteran participants are recruited via three primary avenues: (1) veterans accessing same-day services, (2) veterans with recent documented suicide ideation or behavior, and (3) VHA clinician referrals. Veterans may also self-refer. Usual care for any veteran accessing same-day services involves a diagnostic interview with a triage provider and a psychiatric evaluation, which includes the VHA system-wide standardized Comprehensive Suicide Risk Evaluation (CSRE) and, as indicated, referrals to outpatient mental health services following evaluation. VHA clinicians are required to screen veterans yearly for suicide risk via the C-SSRS screener [21,25] and document this in the veteran’s electronic health record. The C-SSRS screener is also used by VHA clinicians as clinically indicated and is required upon transitions of care (eg, discharge from inpatient psychiatry).

Research screening is used to confirm eligibility. Research staff will be available on the same day to accept a “warm handoff” for screening as needed. For all veterans contacted for screening, a CSRE will be conducted subsequently by the on-call psychologist if their risk level has changed since their last documented CSRE. If the veteran is deemed to be at moderate or high risk, a suicide safety plan will be completed or reviewed and updated. If the veteran is eligible, they will be invited to participate in a separate informed consent meeting and then subsequently enrolled in the study. Following enrollment, veteran participants will complete a diagnostic and baseline assessment and then randomized to SUPPORT or enhanced standard care. Those randomized to the SUPPORT condition will be matched with a peer specialist participant based on the peers’ and veteran’s availability. Randomized matching and veteran preference were considered but deemed not to be feasible given the nature of the pilot design. As feasible, veteran preference for identity characteristics such as gender as well as shared lived experience will be considered in matching as is standard practice for VHA peer services. Veterans not eligible at any stage will receive standard mental health care referrals and a study-designed resource packet.

**Figure 1:**
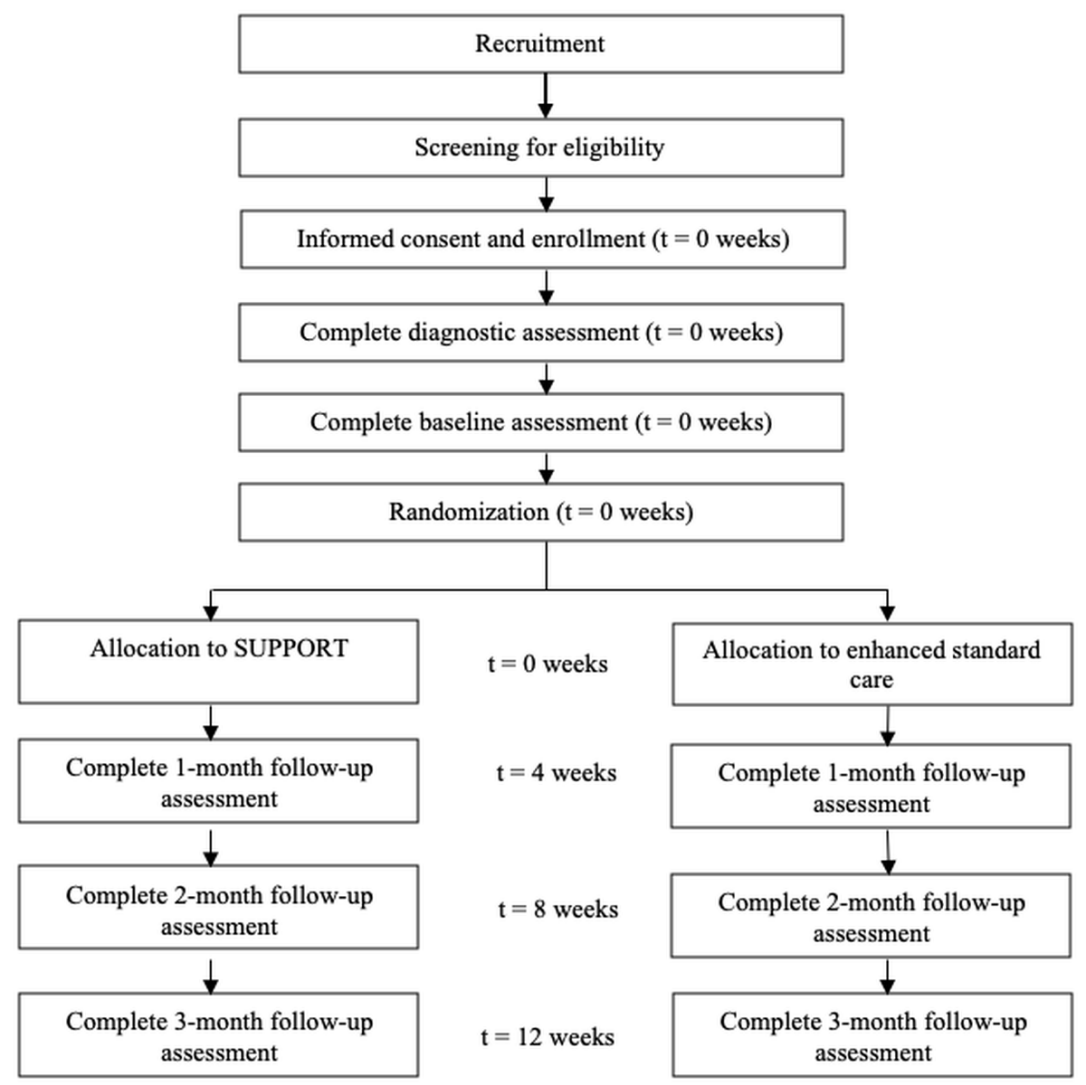
Veteran participant timeline. SUPPORT: Suicide Prevention by Peers Offering Recovery Tactics.

Specific to allocation of conditions, a computer-generated 50-participant randomization table for this study was created and will be maintained by the project’s biostatistician. The table includes no matching on baseline characteristics, 1 follow-up assessment as the minimum number of follow-up assessments needed to be considered as intention to treat, and completion of a minimum of 1 module of the intervention to be considered as intention to treat for those in the SUPPORT condition. After enrollment, the study coordinator emails the veteran participant’s unique identification number to the biostatistician, who then provides the study coordinator with the allocation. There is no masking due to the pilot feasibility and acceptability design. See Figure 1 for the veteran participant timeline flow.

### Safety Procedures

#### Overview

At least one member of the on-call team of 3 licensed psychologists, who are part of the research team and employed at the VA San Diego Healthcare System, is available during all participant interactions. The on-call team is available for suicide risk assessments and appropriate triage as well as support to the research staff and peer specialists. The local suicide prevention coordinator team is also available on call during business hours for additional consultation.

#### Peer Specialist Participants

Peer specialist participants conduct a posttreatment assessment after completing SUPPORT with each veteran participant. To assess potential iatrogenic effects of delivering the intervention, peer specialist participants will complete the Negative Effects Questionnaire [26,27] (See peer specialist participant measure section below) as part of their posttreatment assessment. If the peer specialist participant reports that a negative effect occurred due to delivering the intervention and rate it as “very” or “extremely” impactful, research staff will notify the on-call psychologist to follow up with the peer specialist participant to determine whether additional support, such as referral to the employee assistance program, is needed. Described in more detail in the SUPPORT training section (subsection: Peer Specialist Training), peer specialists also receive ongoing support as part of the study supervision group and can request to meet individually with the principal investigator at any time.

#### Veteran Participants

Veteran participants referred to the study are gauged by a licensed VHA clinician as not needing immediate psychiatric hospitalization; however, as risk can change, veteran participants are assessed for suicide risk at multiple stages of the study. Peer specialists are trained in the SUPPORT safety protocol to directly screen for increasing veteran suicide risk at each SUPPORT appointment [18,28]. This protocol trains the peer specialists to contact the on-call psychologist during the appointment if suicide ideation is reported to be more severe since the last licensed VHA clinician’s assessment and if there is intent to act before the next meeting with a licensed VHA clinician. If suicide ideation is current but reported not to be more severe and it is not likely the veteran will act on their suicide thoughts, peer specialists are to review the veteran’s suicide safety plan and alert the on-call psychologist after the appointment concludes. A detailed procedure for this safety protocol is described in the work by Chalker et al [18].

All veteran participants will also be screened for suicide risk at screening, baseline, and all follow-up assessments by the research staff. Research staff are trained in an adapted version of the University of Washington Risk Assessment Protocol [29,30] to fit VHA protocols. Specifically, research staff are trained to alert the on-call psychologist while still with the participant if they report current active suicide ideation, a plan for suicide, access to lethal means, and intent to act on their plan in the following 48 hours—all of which are questions asked in the baseline and follow-up assessment batteries. If any of the previous criteria or any suicide behavior is reported during baseline and follow-up assessments, research staff are trained to alert the on-call psychologist to review the participant data, and the on-call psychologist will determine the appropriate next steps, for example, contacting the veteran for additional suicide risk assessment. Veterans are informed of limits to confidentiality such that, if they report increases in their acute suicide risk to research staff or peer specialists, their treatment team may be informed by the principal investigator to support continuity of care.

If a veteran participant is psychiatrically hospitalized at the local medical center, research staff and their assigned peer specialist as applicable will coordinate with the veteran’s broader treatment team to determine appropriate ongoing engagement. As part of their role, peer specialists can and do provide services in inpatient psychiatry settings.

### Peer Specialist Training

The SUPPORT training includes two 4-hour workshops tailored to the scope of a peer: SUPPORT 101 (a stand-alone general suicide prevention training) and the SUPPORT intervention training (a training for veteran peer specialists in how to provide the recovery-oriented, evidence-informed intervention for veterans with SMI at increased suicide risk). Training in the SUPPORT safety protocol is also provided. Chalker et al [18] provide a detailed description of the full training.

After the SUPPORT training, a study supervision group is held biweekly. This supervision is led by the principal investigator, who is a licensed clinical psychologist and trained as a peer specialist supervisor in the state of California [31]. Study supervision serves as a dedicated time to discuss immediate concerns; receive feedback on SUPPORT appointments; discuss and process general concerns, fears, and questions; receive support; and discuss implementation or other administrative topics. Peer specialist participants will continue to attend the regular supervision that they receive outside of the study that includes monthly group supervision with all local peer specialists and individual supervision with their direct clinical supervisor.

### SUPPORT Intervention Curriculum

#### Overview

The SUPPORT intervention was co-developed by veterans with lived experience and inspired by 4 evidence-based interventions: suicide safety planning [32,33], Whole Health [34], Wellness Recovery Action Plan [35], and compensatory cognitive training [36,37]. Therefore, the SUPPORT intervention integrates elements of value-based living, recovery and action planning, and compensatory cognitive strategies (called “learning strategies” in SUPPORT). The role of learning strategies is to enhance recall and engagement in plans for adaptive coping during high-risk situations, as well as to enhance recovery plans to work toward meaningful goals [38]. After learning strategies are introduced, the progress of using them is reviewed and updated as needed in subsequent appointments. The content is formatted into 4 modules (Table 1) that are delivered in weekly one-on-one via telehealth or in-person appointments for approximately 50 minutes, each in the usual clinic in which the peer specialists work. The peer specialist conducts brief check-in calls at 2 and 4 weeks after the final module to reinforce the intervention material and offer additional resources as needed. Following the SUPPORT safety protocol, if a veteran is screened to be at increased suicide risk and the on-call psychologist is required to join the appointment, the peer specialist and veteran may reschedule that module’s content to a separate appointment. A SUPPORT intervention note template is provided for peer specialists to document their encounters in the veteran’s electronic health record following their standard clinic procedures.

**Table 1:** Peer-led Suicide Prevention by Peers Offering Recovery Tactics intervention curriculum.

Module number	Theme	Psychoeducation topics	Activities
1	Reasons for living	Recovery, values, and reasons for living	Identifying reasons for living by recognizing strengths and values
2	Hope	Wellness tools and hope kit	Developing wellness tools to feel well, stay well, and live the way they want to; developing a hope kit (a collection of reminders of one’s values, positive memories, and reasons for staying alive)
3	Recovery goal	Recovery goals, action steps, and goal planning	Creating a long-term recovery goal as well as short-, mid-, and longer-term action steps to work toward recovery goals, particularly when suicidal
4	Social connections	Social support and crisis support	Building and maintaining social support, how supports contribute to recovery and suicide prevention, identifying who to call in a suicide crisis, addressing barriers to contacting supports, and reinforcing alternative tools

A workbook is provided to all veterans, which they are encouraged to complete with their peer specialist as well as in between appointments. The prompts in the workbook encourage a conversation focused on recovery and suicide prevention, including building a SUPPORT plan. By the end of SUPPORT, veterans will have a completed SUPPORT plan with 4 reminders for living that map to each module. All SUPPORT appointments also have four objectives for the peer specialist to meet: (1) ask directly about suicide, (2) work on the SUPPORT plan, (3) build learning strategies, and (4) share relevant experiences.

#### Fidelity

All SUPPORT appointments are recorded, and adherence is coded using the SUPPORT fidelity measure—developed with input from both advisory boards who co-developed SUPPORT [18] and based on previous peer specialist fidelity work [39]. Adherence is evaluated at the appointment level. An adherent SUPPORT appointment is defined as meeting all 4 objectives. Given the pilot nature of the trial and development stage of the intervention, all recorded appointments will be coded for adherence by at least one coder to provide feedback to the peer specialist. Adherence ratings are shared weekly with peer specialists and discussed in the study supervision group; peer specialists can meet individually with fidelity coders or the principal investigator as needed. A total of 15% of all SUPPORT appointments will be randomly selected and rated by 2 coders to determine fidelity. If the 2 coders disagree about whether an appointment has reached adherence, a third coder will rate the appointment, and a consensus meeting will be held among the 3 coders.

### Enhanced Standard Care

SUPPORT will be compared to an enhanced standard of care delivered at the VA San Diego Healthcare System that contains the following elements: (1) suicide risk assessment, (2) VHA safety planning intervention, and (3) timely referral to VHA mental health outpatient care. Standard care is said to be enhanced as veterans randomized to this condition will also be assessed for suicide risk at baseline and follow-up assessments, which may result in additional clinical interactions.

### Data Collection and Management

#### Overview

To become an approved assessor, training includes live training by the principal investigator and online training as provided by certain measures such as the C-SSRS [40]. Assessors then sequentially conduct role-plays with approved assessors, shadow an approved assessor, and are finally observed and approved by a previously approved assessor. Data are collected by approved assessors and stored in a VHA-approved secure web application for building and managing online surveys and databases called REDCap (Research Electronic Data Capture; Vanderbilt University) [41,42]. REDCap has validity checks for individual variables (eg, whether the data are in the appropriate range) included in the project design. Once data are entered into REDCap, they are also checked by a separate research staff member. Laboratory meetings are held weekly to discuss data collection and management questions.

#### Feasibility and Acceptability Data

Feasibility is defined as attainment of recruitment goals (n=50 veterans; n=2 peer specialists) and a ≥70% retention rate for veterans at the end of the intervention. Acceptability for veterans is measured using the Client Satisfaction Questionnaire–8 [43]. While direct suicide prevention interventions have been only shown to be helpful [44,45] and our advisory boards did not anticipate any negative effects for veterans, we do ask 1 open-ended question after intervention completion or dropout to determine whether veterans found the intervention to be harmful. After each veteran participant completes or drops out of the intervention, the following acceptability item is asked of the peer specialist to rate on a 5-point Likert scale (1=not at all, 2=a little bit, 3=somewhat, 4=quite a bit, and 5=very much): “Overall, how satisfied are you with delivering the SUPPORT Intervention?” High satisfaction for peer specialists is defined as “quite a bit” or “very much” satisfied.

#### Peer Specialist Participant Measure

To track potentially negative effects for peer specialists, a revised and shortened version of the Negative Effects Questionnaire [26,27] is administered after each veteran participant completes or drops out of the intervention. The Negative Effects Questionnaire was revised from “receiving” to “delivering” an intervention and includes 8 yes or no items related to possible negative effects one might experience because of delivering the intervention. If any item is endorsed positively, two follow-up questions are asked: (1) “Indicate the severity of your experience (how negative the experience was for you)” (response scale: 0=not at all, 1=slightly, 2=moderately, 3=very, and 4=extremely) and (2) “Do you believe your experience was caused by the intervention you delivered or other circumstances that occurred during the same period as this intervention?” Traditional scoring may not be useful, so the Negative Effects Questionnaire results will be reported as (1) a total score of endorsed negative effects (range 0-8), (2) a total score of severity across all endorsed items (range 0-32), and (3) a total score of the negative effects being caused by the intervention (range 0-8).

#### Veteran Participant Measures

Veteran assessments are conducted at baseline and the 1-, 2-, and 3-month follow-ups. Table 2 shows the veteran participant measures. The measures included assess a range of standard outcomes important in the prevention of suicide, as well as including 2 laboratory-developed measures to explore SUPPORT’s potential unique impact on suicide prevention.

The primary outcome of personal recovery is measured using the 41-item Recovery Assessment Scale, a self-report measure of personal recovery with 5 domains: personal confidence and hope, willingness to ask for help, goal and success orientation, reliance on others, and no domination by symptoms [46]. The other primary outcome is assessed using interviewer -administered assessments of the 19-item Beck Scale for Suicide Ideation [47] for past-week suicide ideation severity and the C-SSRS [21,25] for past-month suicide ideation severity.

**Table 2:** Veteran participant measures at all time points.

Timing and domain		Measure	Outcome type
**All time points**			
	Personal recovery	Recovery Assessment Scale–41	Primary
	Suicide ideation severity	Beck Scale for Suicide Ideation; C-SSRS^a^	Primary
	Psychosocial functioning	Specific Level of Functioning Scale	Secondary
	Quality of life	Brief version of the World Health Organization Quality of Life Scale	Secondary
	Cognitive strategy use	Cognitive Problems and Strategies Assessment	Exploratory
	Perceived burdensomeness and thwarted belongingness	Interpersonal Needs Questionnaire–15	Exploratory
	Hopelessness	Beck Hopelessness Scale	Exploratory
	Suicide behaviors	C-SSRS	Exploratory
	Suicide crisis service use	Chart review	Exploratory
**2-month follow-up only**			
	Peer impact	Laboratory-developed measure	Exploratory
**2- and 3-month follow-ups**			
	Suicide prevention strategy recall (SUPPORT^b^ plan and suicide safety plan)	Laboratory-developed measure	Secondary

^a^C-SSRS: Columbia–Suicide Severity Rating Scale.

^b^SUPPORT: Suicide Prevention by Peers Offering Recovery Tactics.

Secondary outcomes of psychosocial functioning and quality of life are measured using the Specific Level of Functioning Scale and the brief version of the World Health Organization Quality of Life Scale, respectively. The Specific Level of Functioning Scale is a 43-item self-report questionnaire assessing functioning on 4 domains (interpersonal, social acceptance, activities, and work) and has been determined to be the best measure of functioning for adults with schizophrenia to assess real-world functioning in 1 scale [48]. The brief version of the World Health Organization Quality of Life Scale is a 26-item self-report instrument assessing 4 domains of quality of life: physical health, psychological health, social relationships, and environment [49]. Collected only at the 2- and 3-month follow-up assessments, suicide prevention strategy recall (secondary outcome) is measured using laboratory-developed self-knowledge tasks in which veterans are given 10 minutes to say what they remember from each section of their SUPPORT plan (SUPPORT condition only) and suicide safety plan [50]. Each plan is scored based on standard safety planning scoring practices [51,52], with SUPPORT plan scores ranging from 0 to 4 and safety plan scores ranging from 0 to 18.

The exploratory outcomes are cognitive problems and strategy use, burdensomeness and belongingness, hopelessness, suicide behavior, service use, and peer impact. Cognitive strategy use is measured using 30 self-report items on memory and thinking strategies that have been successfully used with a population with SMI and designed to capture changes in the use of compensatory cognitive training skills [37], referred to as “learning strategies” in SUPPORT. Burdensomeness and belongingness, which, when they converge, according to the interpersonal theory of suicide [53], create suicide desire [54], are captured using a 15-item self-report measure. The Beck Hopelessness Scale is a 20-item true-or-false self-report measure that has been shown to be predictive of eventual suicide in psychiatric inpatients [55]. Suicide behavior is measured using the C-SSRS behavior section, and service use (ie, suicide-related emergency department visits and inpatient psychiatry stays, Veteran Crisis Line contacts, and outpatient visits) is captured via chart review. Finally, based on theories related to peer support [56-63] and the Peer Specialist Fidelity Measure–Veteran Version (previously used to establish fidelity in traditional VHA peer support programs [39]), we co-developed with our advisory boards a measure collected posttreatment to evaluate the peer impact of veterans receiving SUPPORT. This measure includes 17 Likert scale–rated items and 14 open-ended questions. Likert scale items are rated from 1 (not at all) to 5 (very much). These items will be summed to obtain a total score; a higher score indicates a higher impact of the peer specialist. Sample Likert scale items include the following: “How much did your peer specialist encourage you to be hopeful?” and “How much did you feel connected with your peer specialist?” Open-ended items are asked to better understand the Likert scale–rated items (eg, “What did you have in common with your peer specialist that helped you connect?”).

### Veteran Participant Sample Size Determination

The sample size for the pilot randomized controlled trial was estimated using a previous method provided by Hedeker et al [64] and the Repeated Measures and Sample Size software. This minimum power estimation is based on a 5% attrition rate per time point, correlation of 0.5% of repeated measures, and medium to large effect sizes. A total sample size of 44 will yield a minimum of 80% power to detect a large effect size, defined as a between-group difference increasing linearly from 0 at baseline to 0.7 SD units at the last time point. A large effect size was chosen as this is a pilot feasibility study and is not intended to be powered to detect a small or medium effect [65].

### Analysis of Outcomes

#### Overview

The primary goal of the pilot randomized controlled trial is to evaluate feasibility and acceptability for a potential efficacy study using recruitment and retention rates and the Client Satisfaction Questionnaire–8. In addition to the primary goal, this study will also assess several primary, secondary, and exploratory outcomes. We are aware that we may be underpowered for the following analyses.

#### Primary and Secondary Outcomes

Primary and secondary outcomes will be tested using mixed-effects models [64,66]. This method allows for the inclusion of participants with missing data and those who dropped out without relying on data imputation. It provides an estimate of the individual variability around the population trend and the variability of the individual intercepts (baseline values) and slopes (changes across time and the correlation between them). A fully saturated treatment by visit model will be used for inference. Covariate structure will be chosen based on the Akaike information criterion. Data will be analyzed for all participants who were randomized for whom we have a baseline assessment and at least one follow-up assessment. We expect the dropout rate to be <10% after the baseline assessment (this dropout rate is considered in our power analysis estimation).

#### Exploratory Outcomes

Due to focus on feasibility, recovery, suicide ideation, and functioning, as well as the low anticipated base rates of suicide behaviors and crisis service use and overall sample size, these analyses are exploratory. The pilot randomized controlled trial exploratory analyses are twofold: (1) to compare SUPPORT to enhanced standard care on cognitive strategy use, thwarted belonginess, perceived burdensomeness, hopelessness, rate of suicide behavior, and rate of crisis service use; and (2) to compare SUPPORT to enhanced standard care on the mediational effect of personal recovery on suicide ideation severity. Peer impact will be reported descriptively for those who received SUPPORT, including a deductive analytic approach [67] of open-ended responses to determine themes.

First, mixed-effects models will be conducted as described for the primary and secondary outcomes. Next, given our small sample size, mediators will be tested in separate models [68,69]. We define treatment mediators as mechanisms through which a treatment might achieve its effects. We will test the products of (1) the *a* path (the independent variable [condition] to the mediator [eg, 2-month follow-up personal recovery]) and (2) the *b* path (the mediator to the dependent variable [3-month follow-up suicide ideation severity]). This procedure is a variation of the Sobel test [70] that accounts for the nonnormal distribution of the *a*-*b* path through bootstrapping procedures. Significant mediation effects are indicated when the 95% CI of the indirect path (*a*-*b*) does not overlap with 0. We will test a full mediation model, with changes in the mediator (assessed at the 2-month follow-up) preceding changes in the outcome variable (assessed at the 3-month follow-up).

## Results

This trial was funded in November 2022. Community-engaged intervention and protocol refinement were completed in January 2025. Data collection for the pilot randomized controlled trial began in January 2025 and is projected to continue until May 2027.

## Discussion

### Anticipated Findings

This study’s primary aims are to evaluate the feasibility, acceptability, and preliminary impact of a novel peer specialist–led suicide prevention program for veterans with SMI at risk of suicide. We hypothesize that SUPPORT will be feasible to deliver, with high satisfaction ratings. It is further hypothesized that SUPPORT, compared to enhanced standard care, will yield greater enhancement of personal recovery and reduction in suicide ideation severity (primary outcomes), as well as enhanced psychosocial functioning, quality of life, and suicide prevention strategy recall. Combining suicide prevention training for VHA peer specialists with the creation of a recovery-oriented suicide prevention intervention curriculum, SUPPORT was designed to meet the unique needs of veterans with SMI. Key considerations for this study include adequate training and ongoing supervision for peer specialists, exploring the impact of the peer-delivered component to further add to the nascent peer-based suicide prevention literature, and determining the impact of peer specialists on recovery outcomes for veterans with SMI at increased suicide risk. As this is the first study to our knowledge examining a VHA peer specialist–led suicide prevention program for veterans with SMI, it will be critical to consider feedback from veteran and peer specialist participants alike to best serve the needs of this population and contribute to the growing body of research.

### Limitations and Future Directions

This study is not without limitations. First, the ongoing implementation of SUPPORT in usual care while determining preliminary feasibility, acceptability, and impact lends itself to unique challenges such as staffing turnover, although this enhances the real-world effectiveness of the program. Second, as described in the work by Chalker et al [18], peer specialists are participants instead of only members of the research team, which proves to have advantages and disadvantages. Advantages include the ability to collect crucial data on what it is like for them to deliver a suicide prevention intervention; however, disadvantages include ongoing stigma of peer specialists resulting in the need to be monitored in their intervention delivery work and inability to monetarily compensate them as participants as they are also VHA employees. Various types of compensation or other incentives should be considered in future peer-related research. In addition, it is not clear whether naturalistic changes through the course of treatment will be distinct from those caused by the augmentation. Future research should explore accommodation for these factors in comparing SUPPORT to treatment as usual inclusive of existing suicide prevention initiatives. Other limitations common in pilot studies include a lack of study power and no time-matched comparison not focused on suicide, such as with the Wellness Recovery Action Plan [35], 2 aspects that could be addressed in a future larger randomized trial.

### Conclusions

Overall, this study presents an important opportunity to learn from a group of peer specialists and veteran participants. Pending outcomes of a larger trial, a goal is to implement SUPPORT in a broader capacity across VHA. By incorporating a curriculum to meet the unique social and cognitive needs of veterans with SMI, this study will fill existing gaps in care in terms of both available recovery-oriented interventions within VHA and within the breadth of existing suicide prevention research initiatives.

## Appendix

Multimedia Appendix 1 World Health Organization trial registration dataset.

## Abbreviations

CSRE: Comprehensive Suicide Risk Evaluation
C-SSRS: Columbia–Suicide Severity Rating Scale
HIPAA: Health Insurance Portability and Accountability Act
REDCap: Research Electronic Data Capture
SMI: serious mental illness
SPIRIT: Standard Protocol Items: Recommendations for Interventional Trials
SUPPORT: Suicide Prevention by Peers Offering Recovery Tactics
VA: Veterans Affairs
VHA: Veterans Health Administration

## Data Availability

The datasets generated or analyzed during this study are not publicly available due to data use agreements in place by the Department of Veterans Affairs. A summary of group findings will be published.
