# Supplementary material for: A Recovery-Oriented Suicide Prevention Program Led by Peer Specialists for Veterans With Serious Mental Illness: Protocol for a Pilot Randomized Controlled Trial
Source: JMIR Res Protoc. 2025 Aug 20;14:e66182. doi: 10.2196/66182 (PMC12409174; doi:10.2196/66182)
Supplement: Multimedia Appendix 1 [file resprot_v14i1e66182_app1.docx]

Multimedia Appendix 1. World Health Organization Trial Registration Data Set

| Data category | Information |
| --- | --- |
| Primary registry and trial identifying number | ClinicalTrials.gov NCT05537376 |
| Date of registration in primary registry | 9, September 2022 |
| Secondary identifying numbers | D4239-W |
| Source(s) of monetary or material support | VA Office of Research and Development  VAResearch@va.gov |
| Primary sponsor | VA Office of Research and Development |
| Secondary sponsor(s) | N/A |
| Contact for public queries | SAC, PhD schalker@health.ucsd.edu |
| Contact for scientific queries | SAC, PhD VA San Diego Healthcare System |
| Public title | A Novel Peer-Delivered Recovery-Focused Suicide Prevention Intervention for Veterans With Serious Mental Illness |
| Scientific title | A Novel Peer-Delivered Recovery-Focused Suicide Prevention Intervention for Veterans With Serious Mental Illness |
| Countries of recruitment | United States |
| Health condition(s) or problem(s) studied | Psychotic Disorders, Bipolar Disorders, Suicidal Ideation, Suicide Attempted |
| Intervention(s) | Experimental: SUicide Prevention by Peers Offering Recovery Tactics |
|  | Control: enhanced standard care |
| Key inclusion and exclusion criteria | Veteran inclusion criteria: active suicide ideation in past month or suicide behavior in past three months; serious mental illness diagnosis; capable of informed consent |
|  | Veteran exclusion criteria: cannot complete the assessment battery; current intoxication requiring immediate detoxification or outpatient plan directed specifically to residential substance use disorder (not mental health) services; imminent psychiatric hospitalization |
|  | Peer specialist inclusion criteria: Certified Peer Specialist employed at VA San Diego Healthcare System; 2 years of experience post certification |
| Study type | Primary purpose: Treatment |
|  | Allocation: Randomized |
|  | Masking: None |
| Date of first enrolment | January 2025 |
| Target sample size | 50 |
| Recruitment status | Recruiting |
| Primary outcome(s) | Personal recovery, suicide ideation severity |
| Key secondary outcome(s) | Psychosocial functioning, quality of life |

**Protocol version:** Version 1: January 30, 2025
